# miMatch: a microbial metabolic background matching tool for mitigating host confounding in metagenomics research

**DOI:** 10.1080/19490976.2024.2434029

**Published:** 2024-11-27

**Authors:** Lei Liu, Suqi Cao, Weili Lin, Zhigang Gao, Liu Yang, Lixin Zhu, Bin Yang, Guoqing Zhang, Ruixin Zhu, Dingfeng Wu

**Affiliations:** a Department of Gastroenterology, The Shanghai Tenth People’s Hospital, School of Life Sciences and Technology, Tongji University, Shanghai, P. R. China; b National Center, Children’s Hospital, Zhejiang University School of Medicine, National Clinical Research Center for Child Health, Hangzhou, P. R. China; c Department of General Surgery, Children’s Hospital, Zhejiang University School of Medicine, National Clinical Research Center for Child Health, Hangzhou, P. R. China; d Guangdong Institute of Gastroenterology; Guangdong Provincial Key Laboratory of Colorectal and Pelvic Floor Diseases; Biomedical Innovation Center, Sun Yat-Sen University, Guangzhou, P. R. China; e Department of General Surgery, The Sixth Affiliated Hospital of Sun Yat-Sen University, Guangzhou, P. R. China; f Shanghai Southgene Technology Co., Ltd., Shanghai, China; g National Genomics Data Center & Bio-Med Big Data Center, Chinese Academy of Sciences Key Laboratory of Computational Biology, Shanghai Institute of Nutrition and Health, University of the Chinese Academy of Sciences, Shanghai, P. R. China

**Keywords:** Metagenomics, microbial metabolic background, propensity score matching, causality

## Abstract

Metagenomic research faces a persistent challenge due to the low concordance across studies. While matching host confounders can mitigate the impact of individual differences, the influence of factors such as genetics, environment, and lifestyle habits on microbial profiles makes it exceptionally challenging to create fully matched cohorts. The microbial metabolic background, which modulates microbial composition, reflects a cumulative impact of host confounders, serving as an ideal baseline for microbial sample matching. In this study, we introduced miMatch, an innovative metagenomic sample-matching tool that uses microbial metabolic background as a comprehensive reference for host-related variables and employs propensity score matching to build case-control pairs, even in the absence of host confounders. In the simulated datasets, miMatch effectively eliminated individual metabolic background differences, thereby enhancing the accuracy of identifying differential microbial patterns and reducing false positives. Moreover, in real metagenomic data, miMatch improved result consistency and model generalizability across cohorts of the same disease. A user-friendly web server (https://www.biosino.org/iMAC/mimatch) has been established to promote the integration of multiple metagenomic cohorts, strengthening causal relationships in metagenomic research.

## Introduction

The human body harbors a highly diverse and abundant microbial community that plays a pivotal role in health and disease.^1^ Metagenomic sequencing, a cornerstone in microbiome research, has uncovered ample evidence of associations between the microbiota and various human diseases.^2–5^ However, the susceptibility of human microbiota to a range of host confounding factors, including genetics, environment, age, and lifestyle habits, gives rise to significant interindividual differences in the microbiota.^6^ This variability frequently eclipses the causal relationships between microbiota and diseases, leading to a pervasive issue of low concordance across microbiome studies.^7^ Therefore, controlling for confounding factors has become an essential prerequisite for ensuring accuracy in metagenomic research, especially when integrating multiple cohorts.^8–11^

Today, causal exploration has become the primary objective of metagenomic research, rather than associations.^12,13^ Effective cohort design, such as twin cohort studies^14^ and time-series sampling cohorts,^15^ is instrumental in reducing the impact of host confounding variables, enabling causal discovery. However, this significantly complicates the cohort construction. In reality, cross-sectional study design featuring diseased patients and healthy controls remains the main approach for metagenomic disease research. By matching controls and cases based on common confounders such as age, sex, and body mass index (BMI), it’s possible to alleviate some of the causal distortions attributable to individual differences. Nonetheless, given the fact that human microbiota is influenced by multifarious sources of variation, including genetics, environment, and lifestyle habits, assembling an ideally matched cohort is extremely challenging.^7^ As such, this necessitates the development of a more convenient and efficient matching strategy.

Research has indicated that metabolic interactions of microbes are major drivers of microbial community assembly,^16–18^ mirroring the composite impact of various host confounding factors. While there is interindividual variation in microbial composition, it’s noteworthy that the core metabolic functions of the microbiome demonstrate remarkable consistency across individuals.^19,20^ Also, the specific functional differences between individuals reflect how microbial communities maintain community structure when faced with immune, environmental, or dietary exposures.^20^ From the perspective of complex systems, systems with similar structures are easier to compare and study for their emergent behaviors.^21^ Consequently, the microbial metabolic background, as a core structure of the microbial system, can be a superior baseline for microbial sample matching, by which it’s possible to directly match control and case subjects even in the absence of numerous host confounding factors.^22,23^

Thus, we developed miMatch, a novel microbial metabolic background matching tool that ensures a balance between the case and control groups in single-cohort studies, thereby mitigating spurious microbiome-disease associations. miMatch enables researchers to construct well-matched cohorts for microbial investigations, facilitating more reliable and reproducible analytical outcomes from their data. For added convenience, researchers can utilize miMatch via our open-access web server at https://www.biosino.org/iMAC/mimatch.

## Materials and methods

### miMatch development and algorithmic details

**Figure 1.**
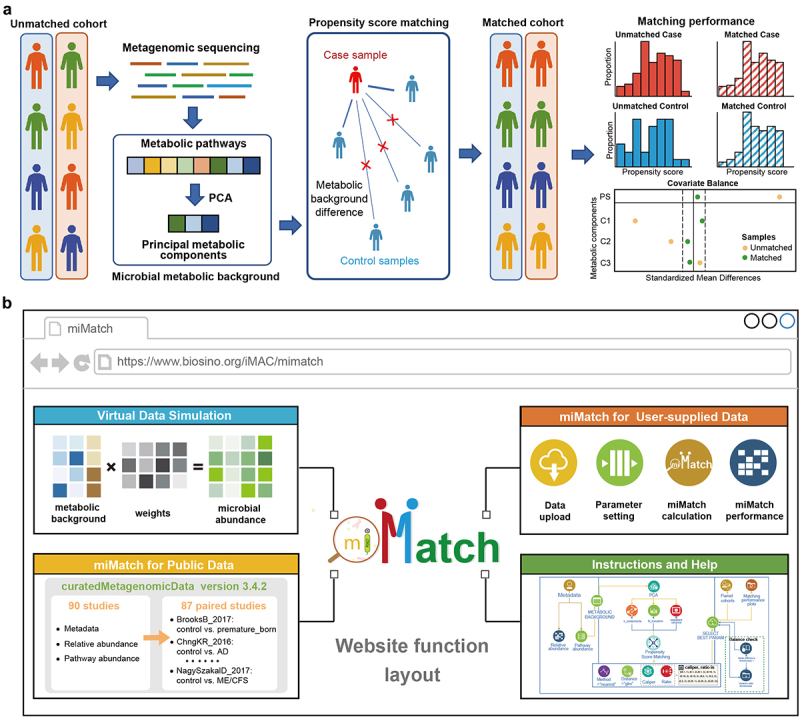
Construction of miMatch. (a) The flowchart of miMatch. For an original unmatched cohort, miMatch first uses principal component analysis to extract key metabolic components from microbial metabolic pathways. Next, propensity-score matching is applied for sample matching. This process constructs a matched cohort by selecting control samples that have similar metabolic profiles to the given case sample. Good matching performance is indicated by a similar propensity score distribution and improved covariate balance between cases and controls. Standardized mean difference measures the difference in propensity scores and key covariates between groups, with values closer to zero (shown by the solid line) indicating better balance. (b) Webpage functional overview of miMatch. PS: propensity score; C1–C3: metabolic components.

To construct a matched cohort from the original cohort, miMatch first uses principal component analysis (PCA)^24^ to extract principal metabolic components from microbial metabolic pathways. Then, based on these metabolic components, propensity-score matching (PSM) is applied for sample matching by the R package “MatchIt”.^25^ The propensity score is calculated using a generalized linear model, and the nearest-neighbor matching algorithm is implemented to search the control sample with the closest propensity score to each case sample, considering all principal metabolic components. The *caliper* parameter specifies a threshold for the maximum propensity score difference of matched pairs. The *ratio* sets the number of control samples matched to each case sample. For example, a *ratio* value of one denotes one-to-one matching. Effective matching is indicated by a similar propensity score distribution and balanced covariate distribution between cases and controls. Covariate balance is examined using the standardized mean difference (SMD), which is calculated by dividing the difference in means of each covariate between groups by the pooled standard deviation. The flowchart of miMatch is shown in Figure 1(a), while the detailed algorithm is illustrated in Figure S1.

### Metabolic background and taxa abundance data were simulated to validate miMatch’s performance

We performed a simulation study using sample sets, each consisting of 100 controls and 100 cases, to investigate the applicability of miMatch. Within a single cohort, the confounding effects between cases and controls were mainly from host interindividual differences. We assumed that the metabolic background is influenced by three representative factors: environment, age, and sex, all following a normal distribution. The metabolic background profile was then generated through random sampling. As illustrated in Figure S2, we defined the microbial abundance in the simulated dataset as the weighted sum of metabolic background and trial. Taxa were classified as differential if their *trial weights* were ≥ 0.1, while taxa with *trial weights* ≤0.001 were classified as other taxa. Specifically, we simulated abundance data of 100 taxa, of which 50 were differential taxa associated with the trial.

We carried out data simulations for three pre-set conditions: 1) the metabolic background was independent of the trial; 2) the metabolic background was affected by the trial through a linear relationship (denoted as *trial effect*); and 3) under the assumption of condition one, the influence of different *trial weight* values on taxa was further tested. For the first condition, the discriminating ability of unmatched and matched cohorts for differential taxa was assessed under varying case-control metabolic background differences, with mean differences ranging from 0 to 0.2 in increments of 0.02. In the second condition, the *trial effect* varied in increments of 0.02 from 0 to 0.1. For both conditions, fifty simulated datasets were generated for each parameter setting, and the average identification accuracy was computed to assess the performance of unmatched versus matched cohorts. Additionally, in condition three, we generated 100 taxa for each *trial weight* value (i.e., 0.001，0.005，0.01，0.04，0.06，0.08, and 0.1) and compared the identification of differential taxa before and after matching. This process was repeated in ten simulated datasets for confidence testing.

### Evaluating the performance of miMatch based on real metagenomic big data

The R package “curatedMetagenomicData” (version 3.4.2) was used to download the human microbiome data of 90 studies, which included metadata, pathway abundance, and taxa relative abundance data. Two studies were excluded due to the lack of pathway abundance information. The remaining studies containing multiple body sites were separated, leading to a total of 101 studies. Since some studies only have cases or controls, we ultimately used 92 studies for further analysis.

We then constructed matched case-control pairs by performing miMatch. Matched case-control cohorts of 87 studies were determined, with 19,060 samples in total. The SMD of microbial metabolic components was used to evaluate the balance after miMatch for each study. The *P*-value and Cohen’s d of the Wilcoxon test were used to compare the differences in microorganisms and pathways between cases and controls. In particular, fold changes were used to describe the degree of change, and *P*-values were adjusted using Benjamini-Hochberg false discovery rate (FDR) correction. A lasso model^26^ was constructed using the Python module “sklearn.linear_model” to assess the effect of each host variable on the metabolic background. Alpha diversity (Faith’s phylogenetic diversity) and beta diversity (PERMANOVA and ANOSIM) were calculated with the Python library “scikit-bio”.

### Assessing miMatch’s performance using consistency evaluation benchmark datasets

To evaluate the result consistency across multiple miMatch-processed cohorts while minimizing the impact of operational and procedural confounding factors, we constructed a set of consistency evaluation benchmark datasets through random subsampling. We selected 61 real-world metagenomic studies, each comprising at least 20 samples per group. For each study, we randomly divided the dataset into two sub-cohorts, and this procedure was repeated ten times for confidence testing, leading to ten sub-cohort pairs. Consequently, a total of 610 sub-cohort pairs were generated and served as benchmark datasets for result consistency evaluation.

The consistency between the two sub-cohorts was assessed by calculating the Spearman correlation of the – log_10_ *P*-value profiles for microbial differences. We evaluated four different analysis strategies: conventional differential analysis (i.e., Wilcoxon rank-sum test) on unmatched cohorts, paired testing (i.e., Wilcoxon sign-rank test) on miMatch-matched cohorts, MaAsLin2 analysis^27^ based on metadata, and MaAsLin2 analysis incorporating miMatch-matched pairs. MaAsLin2 is a statistical method that identifies multivariable associations between clinical metadata and microbial features using generalized linear models.^27^ In the MaAsLin2 analysis, we controlled for key confounders such as age, sex, BMI, disease stage, alcohol consumption, smoking status, and current antibiotic use based on cohorts’ metadata. For the miMatch-based MaAsLin2 analysis, we only used the matched pairs from miMatch as random effects in MaAsLin2 to account for confounding factors.

### Construction and validation of an extended random forest model based on matched samples

To enhance microbial disease diagnostic models by utilizing information from matched samples, we made a simple extension to the Random Forest, a widely used machine learning algorithm. In the conventional construction of Random Forest models, each sub-model uses a random subset of samples to build a decision tree. In our Matched Sampling Random Forest (MSRF), a proportion of the subtrees are built using matched samples (Figure S6A). We can adjust the sample size for each subtree (indicated by parameter N) in MSRF to ensure that the model effectively learns patterns from matched case-control pairs. Theoretically, incorporating matching information may offer benefits, especially when N is smaller and the proportion of the subtrees using matched samples is higher. Despite this modification, MSRF follows the same framework as traditional Random Forest. The Area Under the Receiver Operating Characteristic Curve (AUC) was applied to evaluate the performance of MSRF on validation data.

We assessed the performance of diagnostic models using 61 real metagenomic studies, each consisting of at least 20 samples per group. Multi-cohort validation was implemented for ten diseases, including inflammatory bowel disease and colorectal cancer. For individual study, the samples were randomly divided into two subsets: 60% were used as the training set for model construction, while the remaining 40% were reserved as the test set for model evaluation. For diseases with multiple cohorts, we performed the study-to-study validation, where the model was constructed using one cohort and validated on another. Additionally, we employed a leave-one-dataset-out (LODO) validation approach, which uses all but one cohort to build the model and then validates model performance using the cohort left out.

### Data entry specifications for the miMatch webserver

miMatch webserver (https://www.biosino.org/iMAC/mimatch) has been developed utilizing the “streamlit” (version 1.22.0) Python library and Docker. To initiate the miMatch tool, a user-supplied dataset formatted as a table comprising raw counts is required. The count matrix can be uploaded directly by the user as a text file, which must adhere to the format used by curatedMetagenomicData. Downloadable example datasets are provided for user reference. Moreover, the miMatch tool allows users to personalize their matching process by adjusting parameters such as *ratio* and *caliper*. By adjusting these parameters, users can tailor the matching process according to their specific requirements and research goals.

### Statistical analysis

All statistical analysis was performed using R software (version 4.1.0) and Python software (version 3.9.7). For unmatched data, differences in non-normally distributed variables between cases and controls were tested using non-parametric tests such as the Wilcoxon rank-sum test and the Mann-Whitney’s U test. In contrast, the Wilcoxon sign-rank test was employed to compare cases and controls for the matched data. Correlation analysis via different studies was performed using Spearman’s correlation test. FDR control was done by the Benjamini-Hochberg procedure. Statistical significance was determined using *p* < 0.05.

## Results

### Algorithm overview of miMatch

miMatch is applied to single-cohort studies of whole metagenomic shotgun sequencing (mWGS) data, which provides an accurate panorama of microbial metabolism. miMatch first utilizes principal component analysis (PCA) to extract key metabolic components from microbial metabolic pathways. Then, propensity score matching (PSM) is applied for sample matching based on these metabolic components with empirical parameters. Control samples with a metabolic background (assessed by propensity score) proximate to that of the given case sample are selected for matching. Notably, prior to finalizing the matched cohort, miMatch employs propensity score distribution and covariate balance tests to assess matching performance, allowing for the optimization of matching parameters (Figure 1(a)). For an in-depth understanding of the algorithm process and parameter configuration, please refer to Figure S1. Additionally, researchers can customize parameters and processes through miMatch source codes (https://github.com/dfwlab/miMatch). An open-access web server is also available for online usage (Figure 1(b)).

### Evaluating the performance of miMatch using simulated data

To evaluate miMatch’s performance in terms of identifying differential taxa, we conducted a controlled study using simulated data. Here, we considered the metabolic background components, such as age, sex, and environment, which represent the combined effects of individual confounding factors. Sample grouping was controlled by the *Trial* variable. Species abundance was assumed to be affected by both the metabolic background and trial with random weights (Figure S2).

**Figure 2.**
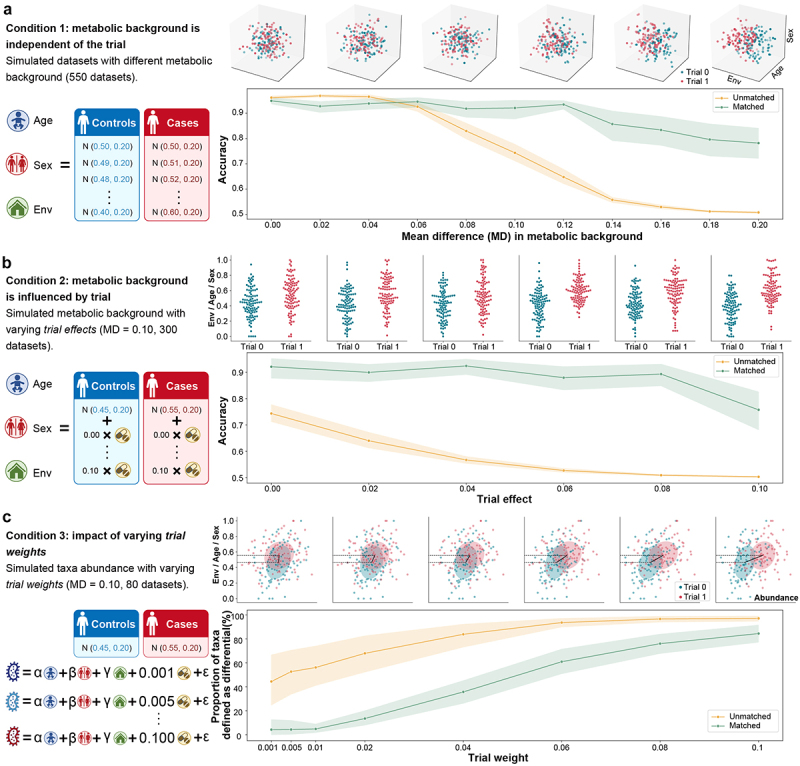
Validation of miMatch’s robustness using simulation datasets under three different conditions. (a) In the first condition, with an independent metabolic background, mean differences (MD) of confounding variables between cases and controls ranged from 0 to 0.2. For each MD value, fifty random datasets were generated for confidence testing, each containing 100 controls and 100 cases (see methods for details). The discriminating ability of unmatched cohorts for differential taxa rapidly declined as MD increased. Example datasets (3D scatter plots) illustrate the distribution differences between trials in the confounding space, with the divergence becoming more pronounced as MD increased. (b) In condition two, the metabolic background was influenced by varying *trial effects* (ranges from 0 to 0.1), with MD fixed at 0.1. A total of 300 datasets were randomly generated. Larger *trial effects* increase distribution differences in confounding factors between groups (strip plots). (c) For the third condition, taxa abundance was generated with varying *trial weights*. Ten simulated datasets were created for each *trial weight* value. Larger *trial weights* lead to more pronounced differences in taxa abundance distribution between trials (scatter plots). Unmatched cohorts were more prone to false positives in differential taxa identification, especially for small *trial weights*. MD: mean difference; Env: environment.

Assuming the independence between metabolic background and trial, we first simulated interindividual differences between groups, with the mean differences (MD) of metabolic background components between cases and controls varying from zero to 0.2 (Figure 2(a), Table S1). The escalating interindividual differences notably disrupted the causality between microbiota and trial. Remarkably, in the absence of confounders (MD = 0), both unmatched and matched cohorts could accurately identify the differential taxa (unmatched = 0.962, matched = 0.949; Figure 2(a), Table S1). In the unmatched cohorts, however, identification accuracy rapidly declined to 0.5 as MD increased, primarily due to an increase in false positives (Figure S3A). In contrast, matching the metabolic background somewhat mitigated the influence of host confounding factors, resulting in significantly higher accuracy (Accuracy >0.9 when MD < 0.14, Figure 2(a)). It should be noted that excessive interindividual differences between trials (MD > 0.14) could further complicate the background matching (*caliper* = 0.20, Figure S3A), thereby impacting the identification of differential taxa (accuracy of matched cohorts decreased from 0.857 to 0.782, Figure 2(a)).

We further simulated a more realistic situation where the metabolic background and trial were not independent (Figure 2(b)). Here, we considered moderate differences in metabolic background between cases and controls (i.e., MD = 0.10) and hypothesized that trial grouping affected the metabolic background through linear interaction. Interestingly, the interaction between trials and metabolic background had a more pronounced effect on the unmatched cohort, with accuracy decreasing from 0.743 to 0.503 (Figure 2(b), Figure S3B, Table S1). This may be due to these interactions confounding the identification of differential taxa between trials. Nevertheless, after applying miMatch for sample matching, matched cohorts demonstrated more robust performance despite a reduction in accuracy (accuracy decreased from 0.921 to 0.757, Figure 2(b), Figure S3B, Table S1).

Under the same confounding influence (MD = 0.10), matched and unmatched cohorts demonstrated distinct results for taxa affected by varying degrees of trials (denoted as *trial weight*, Figure 2(c), Table S2). Compared to matched cohorts, unmatched cohorts showed anomalies in differential taxa identification, especially for taxa with small *trial weights*. Remarkably, even at a *trial weight* of 0.001—indicating virtually identical taxa abundance between trials – nearly 44.40% of the taxa (defined by *p* < 0.05) were identified as differential in unmatched cohorts (compared to only 4.20% in matched cohorts), which could be interpreted as false positives caused by confounding factors (Figure 2(c), Table S2). This conclusion was consistent under different criteria for defining differential taxa (Figure S4).

### The applicability of miMatch in real-world metagenomic studies

**Figure 3.**
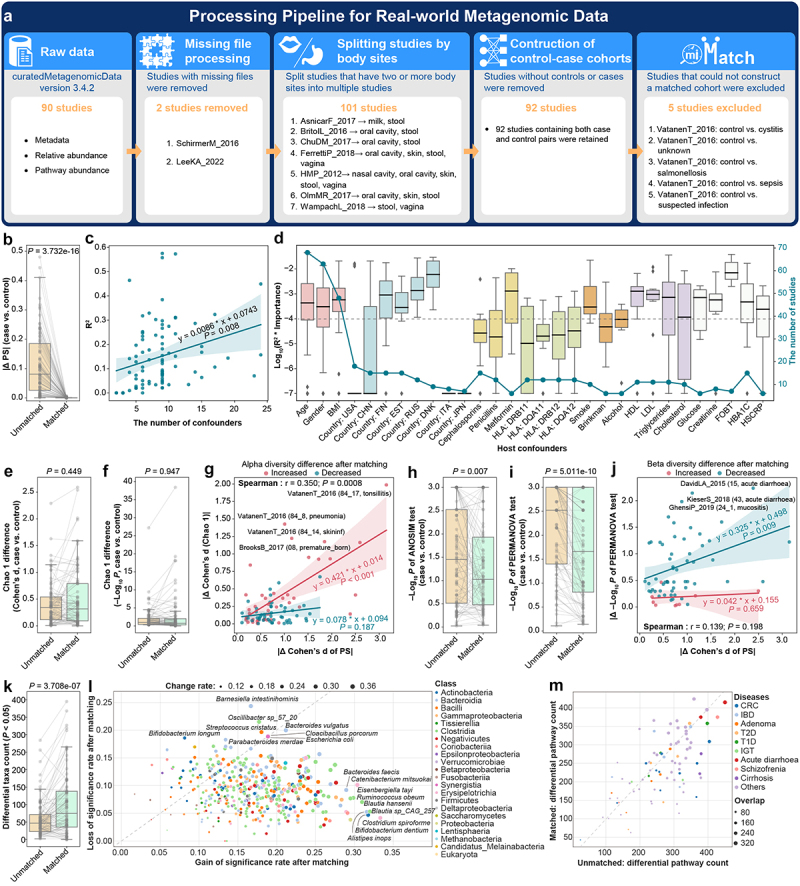
The applicability of miMatch in real-world metagenomic studies. (a) The flowchart outlining the process for filtering real-world data from the curatedMetagenomicdata database. (b) The metabolic background differences between groups were significantly reduced after miMatch matching. (c) Propensity scores can be predicted by host confounding factors, with the number of confounders significantly influencing prediction accuracy (R^2^). (d) The importance (boxplots) and study counts (blue points) for host confounding variables. R^2^ indicates the model’s goodness of fit. The feature importance was measured by randomly permuting the confounder 100 times. (e) and (f) alpha diversity differences between groups (Cohen’s d and –log_10_ *P*-value) were consistent after matching. (g) The increase in alpha diversity differences (red dots) was correlated with changes in propensity score differences after matching. Each dot represents a study. The direction of change in alpha diversity differences is indicated by different colors. (h) and (i) beta diversity differences significantly decreased after matching. (j) The decrease in beta diversity differences (blue dots) was related to changes in propensity score differences after matching. (k) Increased number of differential taxa after miMatch matching. (l) The rate of significance changes for different microbes after applying miMatch. (m) The number of differential metabolic pathways remained similar after matching. Each dot represents a study. The size of each dot, labeled as “overlap”, denotes the number of differential pathways that overlapped before and after matching. Gray lines in panels (b), (e), (f), (h), (i), and (k) connect data from the same studies. PS: propensity score; BMI: body mass index; HBA1C: hemoglobin A1c; HDL: high-density lipoprotein; LDL: low-density lipoprotein; HLA: human leucocyte antigen; FOBT: fecal occult blood test; HSCRP: high sensitivity C reactive protein.

We employed 90 studies with 20,533 samples from the curatedMetagenomicData to systematically evaluate the application of miMatch in real-world metagenomic research (Figure 3(a), Table S3). In most studies, there was a certain degree of metabolic background difference between the case and control groups (average propensity score difference = 0.115, Figure 3(b), Table S3). After processing by miMatch, the metabolic background differences were significantly reduced (average propensity score difference < 0.001, *p* < 0.001, Figure 3(b), Table S3).

The propensity score, as the ultimate representation of each sample’s metabolic background, was calculated solely based on the individual’s gut microbiota metabolic pathway information. Interestingly, we found that the propensity score could be predicted through the linear regression of host confounders, and the prediction accuracy (R^2^) was significantly affected by the number of confounders (coefficient = 0.0086, *p* = 0.008, Figure 3(c)). However, in most studies, the inclusion of host confounding factors still fell short of accurately depicting the variations in metabolic background (R^2^ <0.60). According to the regression feature importance, geographical location, medication (i.e., metformin treatment), lifestyle factors (i.e., smoking and alcohol consumption), and other clinical indicators (i.e., lipoproteins, triglycerides, glucose, creatinine, and fecal occult blood test [FOBT]) also significantly contribute as host confounders, in addition to the commonly used age, sex, and BMI in microbiome studies (Figure 3(d)).

In most metagenomic studies, the alpha diversity differences between cases and controls remained similar after matching the metabolic background (*p* > 0.05, Figure 3(e-f), Figure S5A, Table S3). In a few studies, such as VatanenT_2016,^28^ the significant increase in alpha diversity differences was related to the change in propensity score differences after miMatch matching (coefficient = 0.421, *p* < 0.001, Figure 3(g)). Notably, multiple studies constructed by VatanenT_2016 were excluded from the analysis due to excessive differences that made matching more difficult (Figure 3(a)). In contrast, the case-control beta diversity differences significantly decreased after matching (ANOSIM: *p* = 0.007, PERMANOVA: *p* < 0.001, Figure 3(h-i), Figure S5B), particularly in studies related to acute diarrhea (Figure 3(j)).

Overall, the majority of metagenomic studies identified more differential microbes after matching (*p* < 0.001, Figure 3(k), Figure S5C). Microbes such as *Barnesiella intestinihominis* were identified as differential in many studies before matching but not after (19/78 = 24.36%), while the differences in some microbes, such as *Clostridium spiroforme* (24/72 = 33.33%), *Blautia sp_CAG_257* (24/75 = 32.00%), and *Bifidobacterium dentium* (27/85 = 31.76%) tended to be overlooked under unmatched metabolic background conditions (Figure 3(l)). Moreover, although matching the metabolic background eliminated metabolic differences to a certain extent, the number of differential metabolic pathways between cases and controls did not change significantly after matching (Figure 3(m)). There were, of course, some variations across different studies and in their respective methods of evaluating differences (Figure S5D).

### miMatch benefits the consistency in metagenomic research

**Figure 4.**
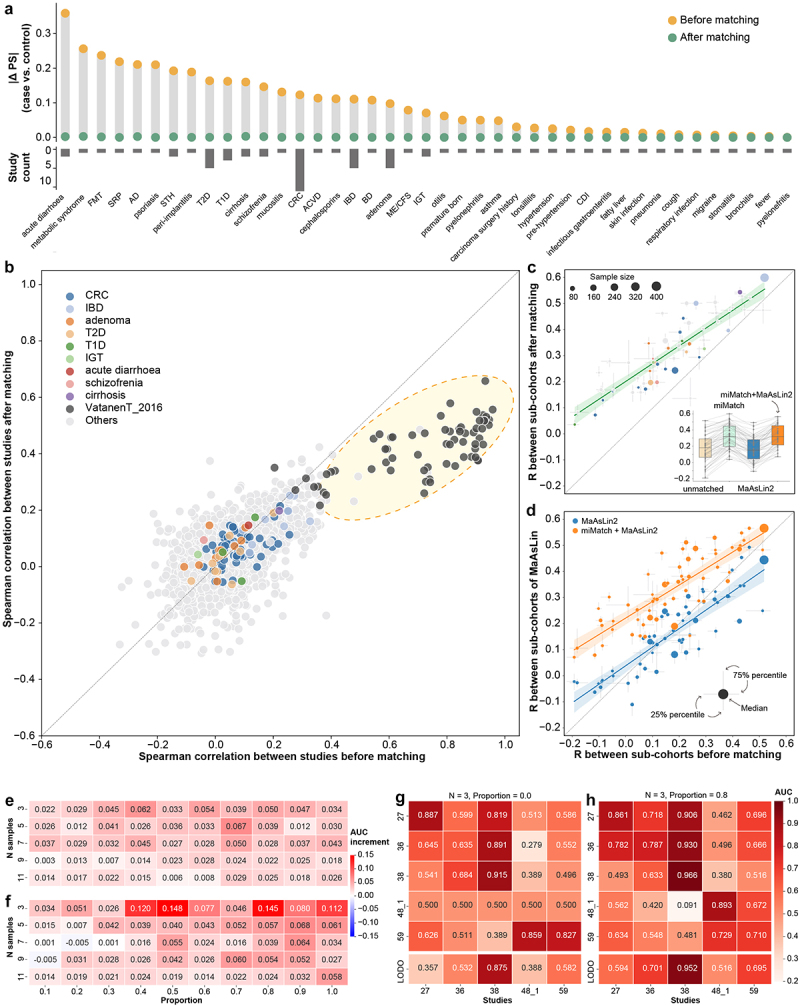
The impact of microbial metabolic background imbalance on diseases. (a) Propensity score differences between groups in both unmatched and matched cohorts for different diseases. The number of studies is shown. (b) Comparison of Spearman correlation across 87 studies before and after matching. Regardless of matching, correlations of microbial variations between groups (–log_10_ *P*-value) across studies were minimal. Studies from the VatanenT_2016 project exhibited unusually high inter-study similarities. (c) and (d) display the Spearman correlation results for consistency benchmark datasets under four conditions: before matching, after miMatch matching, using MaAsLin2, and using MaAsLin2 incorporating miMatch pairs. Each dot represents a study. (e) and (f) display average AUC increment for IBD diagnostic models in study-to-study (e) and leave-one-dataset-out (LODO, F) validation, respectively. Different combinations of sample size per subtree and proportions of the subtrees using matched samples were tested. (g) and (h) display the AUC validation matrices for IBD diagnostic models, where (g) represents models without matched samples and (h) represents models with 80% of the subtrees using matched samples. In the study-to-study validation, models were trained on the cohort listed on the y-axis and tested on the cohort listed on the x-axis. For LODO validation, models were trained on all studies except the one being tested. Diagonal AUC values in the study-to-study validation were computed using a training-test split. Detailed project information related to these models is provided in table S3. FMT: fecal microbiota transplantation; AD: Alzheimer disease; STH: soil-transmitted helminth; T2D: type 2 diabetes mellitus; CRC: colorectal cancer; T1D: type 1 diabetes mellitus; ACVD: atherosclerotic cardiovascular disease; IBD: inflammatory bowel disease; BD: behcet’s disease; ME/CFS: myalgic encephalomyelitis/chronic fatigue syndrome; IGT: impaired glucose tolerance; CDI: *clostridioides difficile* infections.

Many studies were affected by the microbial metabolic background, especially in cases of acute diarrhea, metabolic syndrome, and fecal microbiota transplantation (FMT) (Figure 4(a)). The impact of metabolic background was particularly evident in microbial beta diversity, with differences between cases and controls substantially diminished after matching (Figure S5E, F). Whether studies were matched or not, correlations in case-versus-control microbial differences across different disease studies were slight (unmatched: average *r* = 0.066, matched: average *r* = 0.058, Figure 4(b)). Host confounding variables, which could skew microbial variations, led to abnormally high inter-study similarities in some studies, such as those in the VatanenT_2016 project, before matching (unmatched: average *r* = 0.724). Matching based on metabolic background could effectively address these spurious correlations caused by confounding effects (matched: average *r* = 0.417, Figure 4(b)).

Given that variations in methodological factors (e.g., sampling, extraction methods, sequencing techniques, and analysis pipelines) and study design (e.g., inclusion and exclusion criteria of subjects) can influence cross-cohort consistency, we constructed a consistency evaluation benchmark by splitting real-world metagenomic studies. Results from miMatch-processed cohorts demonstrated a significant improvement in result consistency between sub-cohorts, particularly in cases where consistency was low before matching (Figure 4(c)). In contrast, MaAsLin2, a representative microbial analysis tool, underperformed, likely due to the lack of key confounding variables in the metadata (Figure 4(d)). Interestingly, miMatch may substitute for unobserved host confounding variables, thereby enhancing the performance of MaAsLin2 (Figure 4(c,d)).

### miMatch improves the accuracy of disease diagnosis

Microbes have emerged as one of the critical biomarkers for diagnosing human diseases. We discovered that even with the simplest model design (Figure S6A), miMatch could provide substantial benefits to disease diagnostic models based on metagenomics (Figure 4(e-h), Figure S6B-F). Within the framework of the Random Forest model, we replaced random samples in a certain percentage of subtrees with matched samples (namely Matched Sampling Random Forest, MSRF), enabling the model to capture the matching information (Figure S6A). Overall, the integration of matching information from miMatch improved the performance of the disease diagnostic model within the single cohort (Figure S6B), with the best-performing study showing an AUC increment of up to 0.46 (cohort 37_1, impaired glucose tolerance, Figure S6C).

Metagenomic studies have consistently grappled with issues of low concordance across studies.^7^ Our findings indicated that miMatch could improve the generalizability in multi-cohort modeling (Figure 4(e-h), Figure S6D-G). For instance, in the study-to-study validation, the model’s AUC showed an average increase of 0.067 for inflammatory bowel disease (IBD, *N* = 5, Proportion = 0.7, Figure 4(e)) and 0.021 for colorectal cancer (CRC, *N* = 3, Proportion = 0.8, Figure S6D). In the leave-one-dataset-out (LODO) validation, the benefits of miMatch’s matching data for cross-study extrapolation capabilities were even more significant, with the model’s AUC rising by an average of 0.148 for IBD (*N* = 3, Proportion = 0.5, Figure 4(f)) and 0.070 for CRC (*N* = 3, Proportion = 0.8, Figure S6E).

### miMatch facilitates meta-analysis of multiple cohorts: a case study on IBD

**Figure 5.**
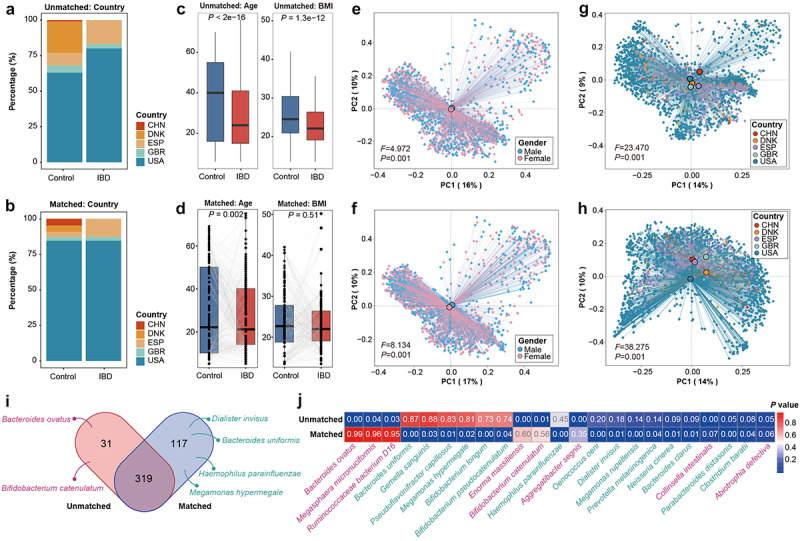
Impact of confounding variables on microbial variations in a case of IBD. (a) and (b) compare the country distribution between cases and controls in five IBD studies, before and after miMatch matching, respectively. miMatch balanced the country distribution between two groups. Noted that there were no IBD samples from China (CHN) or Denmark (DNK) in the original cohorts. (c) and (d) compare the distribution of age and BMI before and after matching. miMatch reduced differences in these confounding variables between groups. (e–h) microbial diversity differences between genders and across countries were significant (e, g) before and (f, h) after matching. The median PERMANOVA *P*-value and F statistic are shown in the plots. (i) A large proportion of differential taxa were retained after matching, with 117 new differential taxa identified. (j) Comparison of *P*-values for previously reported ibd-associated species between unmatched and matched cohorts. Differential taxa unique to the unmatched cohort are highlighted in red, while those only reported in the matched cohort are marked in green. CNH: China; DNK: Denmark; ESP: Spain; GBR: Great Britain; USA: United States of America.

miMatch can also aid in the meta-analysis across multiple cohorts. Taking IBD research as an example, we compiled data from five IBD studies, encompassing 796 control samples and 1730 IBD samples. After metabolic background matching by miMatch, there were 2023 paired control-IBD samples obtained. Factors such as country, age, and BMI have been considered significant host confounders in IBD research.^29^ While miMatch was employed to match the microbial metabolic background of the host, we observed that the balance between controls and cases in terms of country, age (unmatched: *p* < 0.001, matched: *p* = 0.002), and BMI (unmatched: *p* < 0.001, matched: *p* = 0.510) was concurrently achieved (Figure 5(a-d)). Furthermore, miMatch only controlled sample pairing and did not affect the microbial composition. As a result, microbial diversity differences across gender (unmatched: *F* = 4.972, *p* = 0.001, matched: *F* = 8.134, *p* = 0.001), country (unmatched: *F* = 23.470, *p* = 0.001, matched: *F* = 38.275, *p* = 0.001), and group (unmatched: *F* = 8.971, *p* = 0.001, matched: *F* = 14.206, *p* = 0.001) were still evident (Figure 5(e-h), Figure S7). Based on the results from miMatch, statistical tools such as the Wilcoxon signed-rank test can effectively utilize pairing information to eliminate the influence of host confounders on real disease-associated microbial species.

We analyzed and compared the differences between cases and controls using the Wilcoxon rank-sum test and the Wilcoxon signed-rank test for matched and unmatched cohorts, respectively (Table S4). After miMatch matching, a significant proportion of differential taxa were retained (319 taxa, Figure 5(i)). However, 31 differential taxa were no longer detected after matching, including eight previously recognized IBD-associated species; 117 new differential taxa were uncovered, of which 15 had been related to IBD in the literature (Figure 5(i, j)). Notably, *Bacteroides uniformis* and *Haemophilus parainfluenzae* have been reported to differ between Crohn’s disease (CD) first diagnosis samples and controls,^30^ and *Dialister invisus* also characterizes dysbiosis in CD samples.^31^ Intracolonic bile acids also have a pivotal role in governing gut inflammation, with an observed positive correlation between *Megamonas hypermegale*.^32^ None of these species was found in the unmatched cohort’s differential taxa.

## Discussion

As is well known, the human body harbors a highly diverse and abundant microbial population. A plethora of evidence suggests that compared to human genetic factors, the microbiome can explain a greater percentage of phenotypic variation for specific diseases in the population,^33^ serving as a novel biomarker for disease diagnosis or treatment. However, the microbiota is highly specific and influenced by various factors such as genetics,^34^ environment,^35^ antibiotic usage,^36^ dietary habits, and lifestyle.^37^ Thus, host confounding variables may obscure the true relationship between the microbiota and disease in current research. In this study, we have developed a novel microbial matching tool called “miMatch”, which can balance the metabolic background between cases and controls, thereby avoiding spurious associations between microorganisms and human diseases. Moreover, miMatch is a publicly available standalone platform that offers user-friendly webpages that enable researchers to use our tool on their metagenomic cohorts.

Propensity score matching (PSM) is a commonly used method in clinical research for controlling confounding factors and enhancing causal inferences.^38,39^ However, the reporting quality of current studies that use PSM remains suboptimal, and constructing a comprehensive host confounding factor-matching cohort poses challenges.^40^ The reality is that a multitude of host confounding factors are often not fully captured during the cohort construction. Specifically, beyond the commonly utilized factors in traditional matching methods, such as sex, age, and BMI, our study underscores the importance of considering other host confounders. These include geographic location, medication (e.g., metformin), lifestyle (e.g., smoking and alcohol consumption), and clinical indicators (e.g., fecal occult blood test [FOBT]), which are often neglected in metagenomics research.^7^

In line with the study by Ivan et al.,^7^ geographic location, alcohol consumption, and stool quality (indicated by the FOBT in our study) are the most significant generalized sources of heterogeneity in human gut microbiota profiles. We also observed the influence of host confounding factors on conditions such as type 2 diabetes, respiratory diseases, and inflammatory bowel disease. Furthermore, we found that studies on acute diarrhea, metabolic syndrome, and fecal microbiota transplantation are more susceptible to the effects of host confounders and require extra caution during the research process. Due to the limited number of cohorts and the heterogeneity across studies, the statistical findings in real-world metagenomics are preliminary and require further validation.

The lack of host confounding factors complicates the construction of matched cohorts, limiting the effectiveness of methods such as MaAsLin2.^27^ By matching the metabolic background, miMatch can integrate a wide range of potential confounders into the matching process, improving the accuracy of microbiome-based disease association studies. Additionally, miMatch-processed cohorts can be seamlessly integrated with microbial analysis tools like MaAsLin2, enhancing their performance.

Metagenomic studies have consistently faced the issues of low concordance, with the influence of host confounding factors being one of the primary reasons.^7^ miMatch is designed to mitigate confounders between cases and controls in single-cohort studies. However, beyond host confounding factors, methodological-related factors and study design factors – which lie outside miMatch’s intended scope – also play substantial roles in influencing result consistency across metagenomic studies. While miMatch cannot fully eliminate these batch effects across cohorts, enhancing result accuracy within each cohort contributes to the conclusion consistency. Results from the consistency evaluation benchmark further demonstrated that miMatch can effectively improve result consistency across different cohorts when research conditions are similar. Meanwhile, miMatch impressively enhances the cross-cohort accuracy of microbial disease diagnostic models and strengthens the effects of meta-analysis. Even in the simplest model design, the introduction of matching information can significantly improve model performance. Therefore, as metagenomic data accumulates, miMatch will become progressively essential for big data integration.

It should be noted that miMatch cannot guarantee the successful construction of matched pairs when there is significant divergence in the primary metabolic background between cases and controls. Researchers should carefully consider this issue when designing experiments. Similarly, miMatch matching may lead to the elimination of some samples, with a resultant loss of valuable information. Therefore, miMatch is not recommended for sample matching in cohorts where confounding factors have been adequately controlled. Moreover, due to the absence of machine learning algorithms specifically designed for matched samples, the full benefits of miMatch for microbial disease diagnosis cannot be entirely realized. A well-designed machine learning algorithm is a prerequisite for miMatch to fully manifest its potential.

## Conclusions

In this study, we introduced miMatch, a web server that provides users with a tool for building metagenomic matching cohorts for free. By rigorously matching the microbial metabolic background between cases and controls, miMatch can improve the consistency of human disease microbiome studies and accelerate the understanding of microbial involvement in pathogenesis. With the ability to avoid spurious microbial associations with human diseases, miMatch can also enhance the standardization in metagenomics research and facilitate causal exploration.

## Supplementary Material

Supplemental Material

## Data Availability

The human microbiome data was downloaded using the R package “curatedMetagenomicData”. All the software packages used in this study are open-source and publicly available. The simulated datasets and code used and/or analyzed in this study are available on GitHub at https://github.com/dfwlab/miMatch.

## List of abbreviations

PSM: propensity score matching
IBD: inflammatory bowel disease
CRC: colorectal cancer
PCA: principal component analysis
FDR: false discovery rate
BMI: body mass index
SMD: standardized mean difference
PERAMANOVA: permutational multivariate ANOVA
ANOSIM: analysis of similarities
MSRF: matched sampling random forest
AUC: the area under the receiver operating characteristic curve
mWGS: whole metagenomic shotgun sequencing
