# Supplementary material for: miMatch: a microbial metabolic background matching tool for mitigating host confounding in metagenomics research
Source: Gut Microbes. 2024 Nov 27;16(1):2434029. doi: 10.1080/19490976.2024.2434029 (PMC11610556; doi:10.1080/19490976.2024.2434029)
Supplement: Supplemental Material [file KGMI_A_2434029_SM5999.zip › KGMI 2434029 Supplementary/Supplementary.docx]

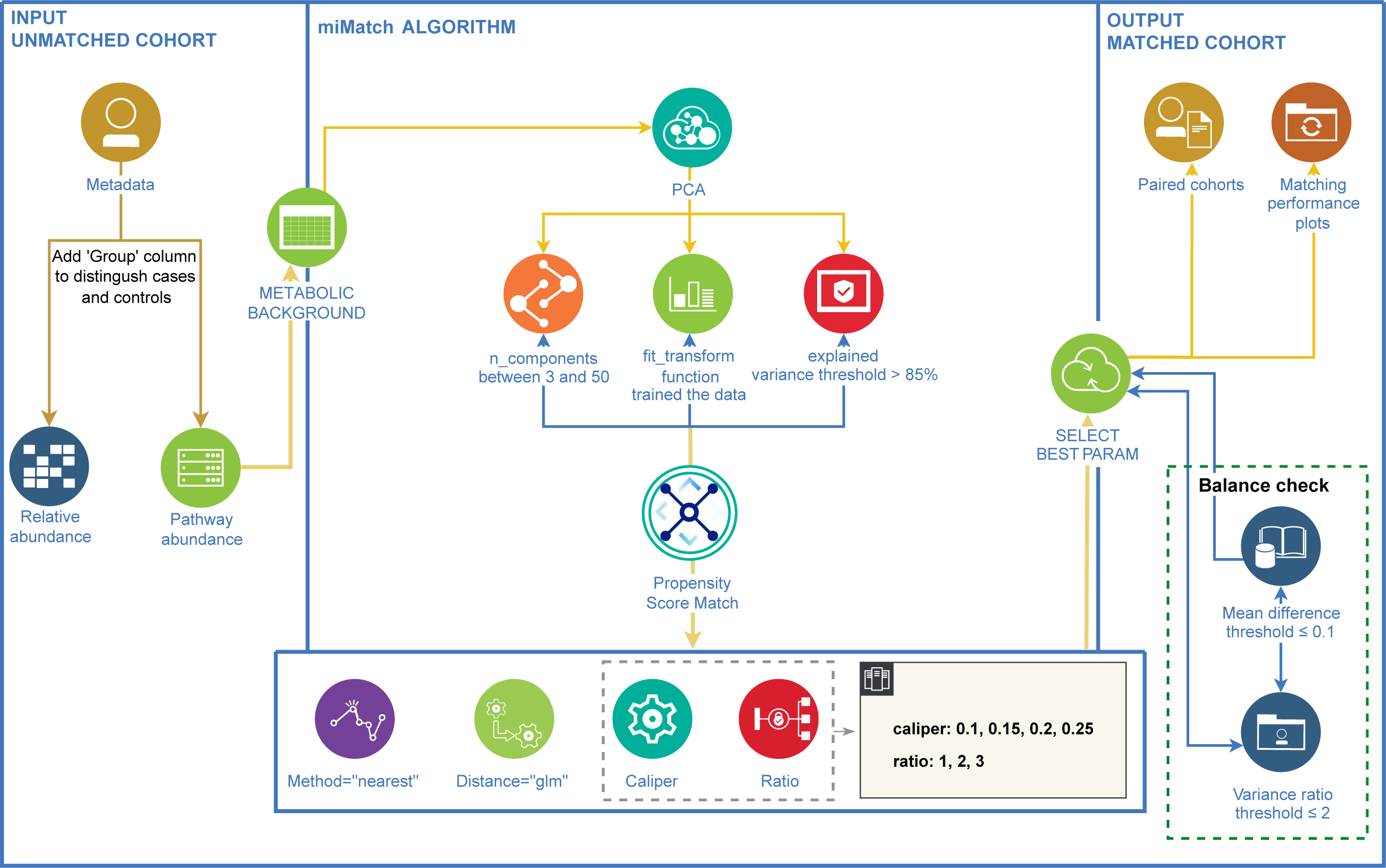


**Figure S1. Flowchart of the miMatch algorithm.** Principal component analysis (PCA) is used to extract principal metabolic components from microbial metabolic pathways with the “sklearn” Python library. The *n_components* parameter specifies the number of components to retain, while the *fit_transform* function trains the data and keeps components explaining over 85% of the variance. Subsequently, propensity-score matching is performed using the “MatchIt” R package. Propensity scores are estimated via a generalized linear model, and nearest-neighbor matching is implemented. Case-control pairs with propensity score differences greater than the *caliper* are excluded from matching. The *ratio* parameter determines the number of control samples matched to each case subject. Matching performance is assessed by evaluating the propensity score distribution and the standard mean differences between cases and controls.


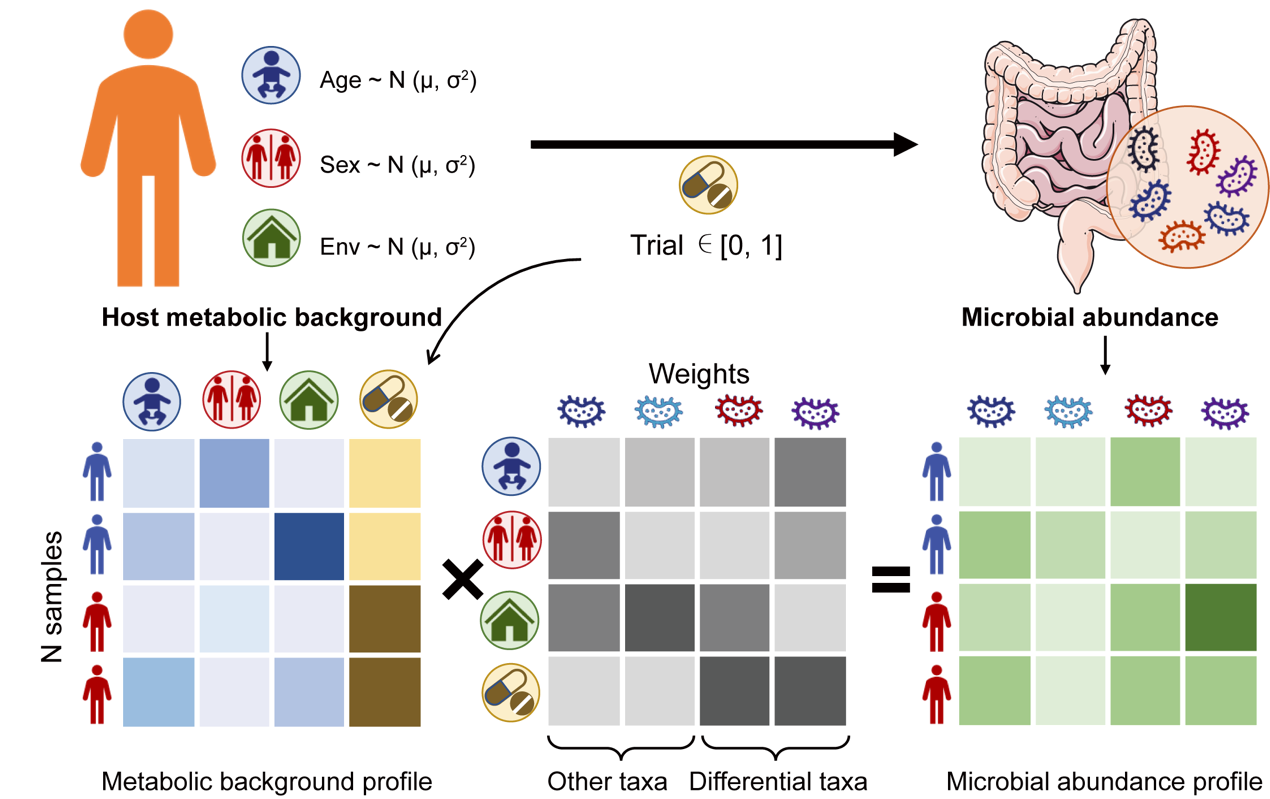


**Figure S2. Schematic diagram of the simulated dataset.** The data simulation models metabolic background components (i.e., age, sex, and environment) as normally distributed. Sample grouping is determined by the *Trial* variable: control samples have a *Trial* value of zero, while case samples have a value of one. Microbial weights are assumed to be affected by both metabolism and trial, with weights generated randomly. Taxa are classified as differential if their trial weights are ≥ 0.1, and as other taxa if their trial weights are ≤ 0.001. Each dataset includes 100 simulated taxa, with 50 designated as differential taxa.


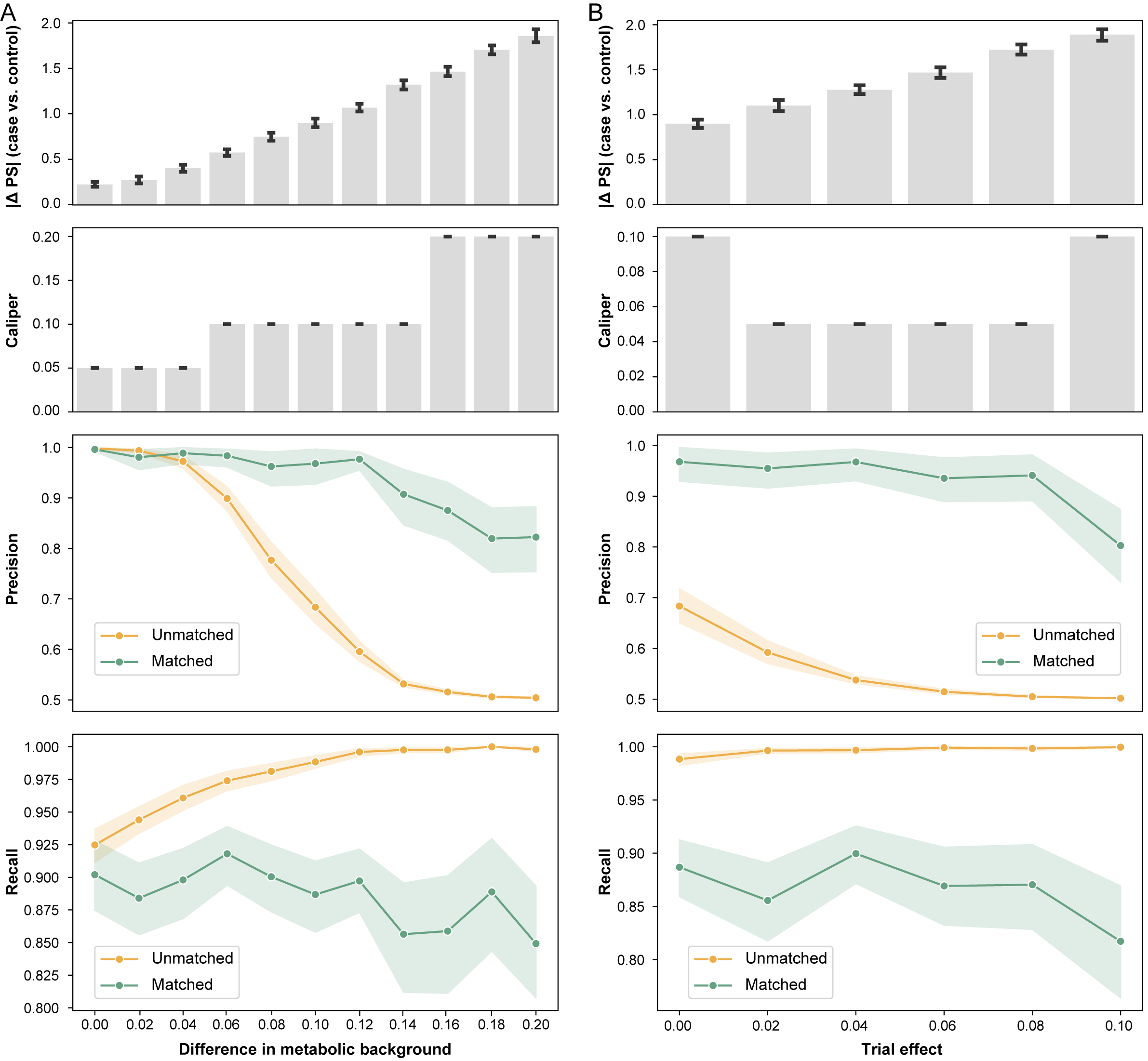


**Figure S3. Performance of miMatch on simulated datasets.** This figure displays miMatch performance for two conditions: (A) the metabolic background was independent of the trial, and

(B) the trial affected the metabolic background. The figures show distributions of propensity score differences between cases and controls, *caliper*, precision, and recall. In unmatched cohorts, the incidence of false positives rose with greater mean differences in metabolic background between cases and controls or stronger trial effects on metabolic background. PS: propensity score.


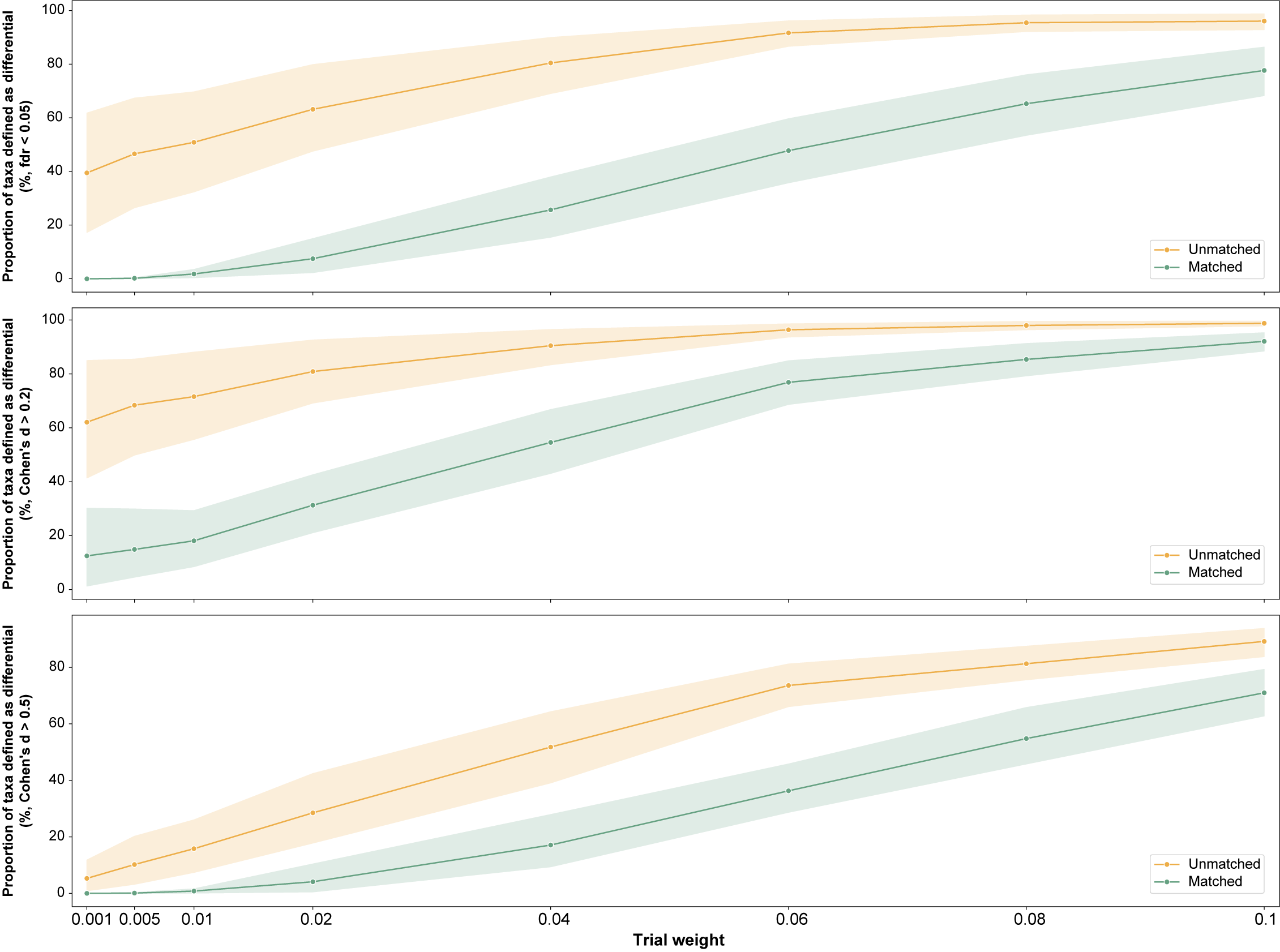


**Figure S4. The proportion of taxa classified as differential under different criteria as trial weights vary.** Differential taxa were identified using a false discovery rate (FDR) threshold of < 0.05, Cohen’s d with a threshold of 0.2, and Cohen’s d with a threshold of 0.5. Compared to matched cohorts, unmatched cohorts were more likely to generate false positives, especially for taxa with small trial weights.


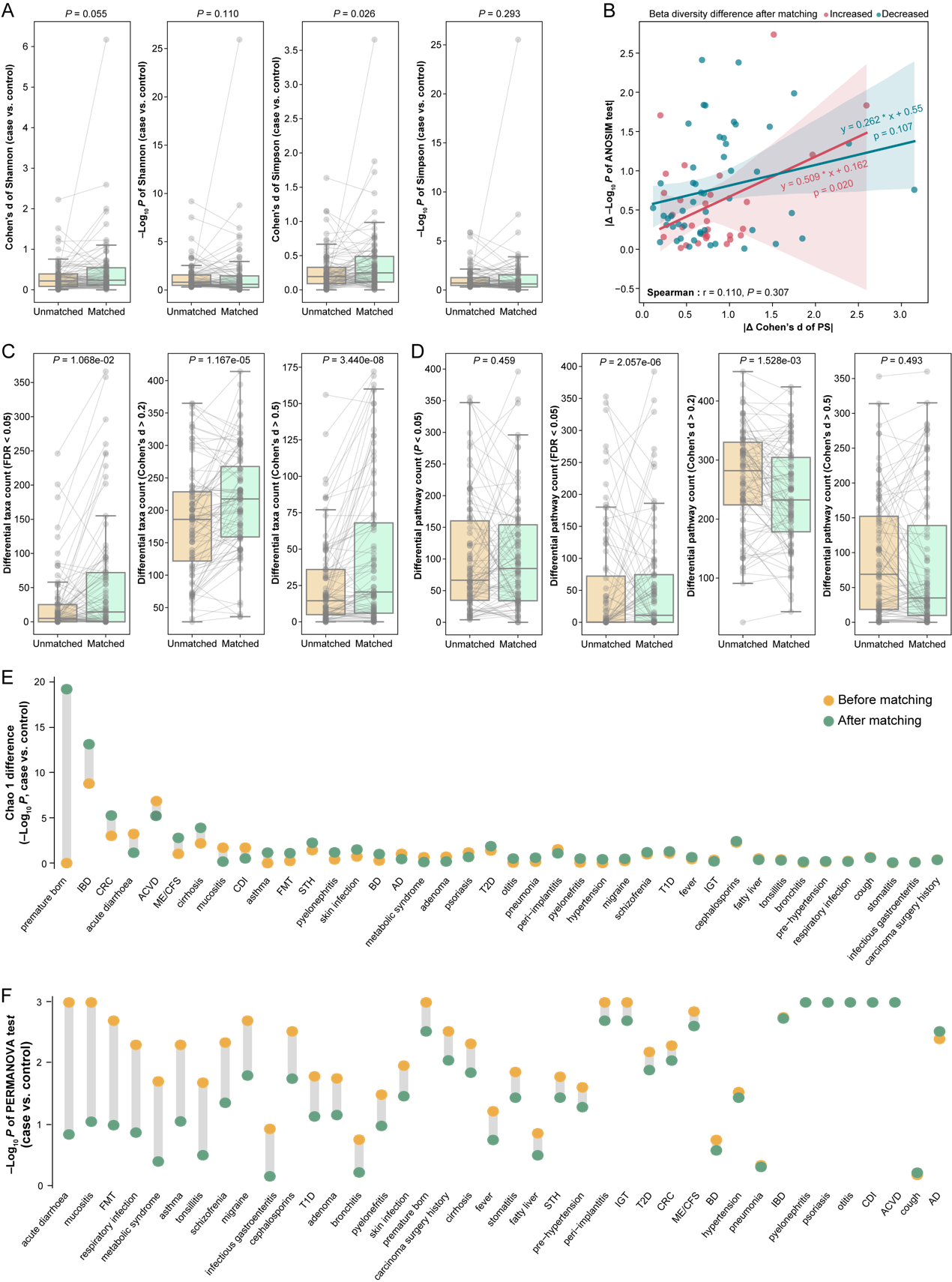


**Figure S5. Case-versus-control differences in real metagenomic data before and after matching.** (A) Alpha diversity differences between cases and controls remained consistent after matching across most studies. Shannon and Simpson indices were used to estimate alpha diversity and different significance test methods were implemented. (B) The relationship between changes in beta diversity differences and changes in propensity score differences between the case and control groups after matching. Red dots represent studies where the case-versus-control difference in beta diversity increased after matching, while blue dots denote studies with a decreased difference. (C) The number of differential taxa increased after matching, irrespective of the

identification criteria used. (D) As the identification criteria changed, the estimated trend in the number of differential metabolic pathways after matching fluctuated. (E) and (F) display the case-versus-control differences in alpha diversity and beta diversity before and after matching for different diseases. The same studies are linked by gray lines in panels (A), (C), and (D). FMT: fecal microbiota transplantation; AD: Alzheimer disease; STH: soil-transmitted helminth; T2D: type 2 diabetes mellitus; CRC: colorectal cancer; T1D: type 1 diabetes mellitus; ACVD: atherosclerotic cardiovascular disease; IBD: inflammatory bowel disease; BD: behcet's disease; ME/CFS: myalgic encephalomyelitis/chronic fatigue syndrome; IGT: impaired glucose tolerance; CDI: *clostridioides difficile* infections.


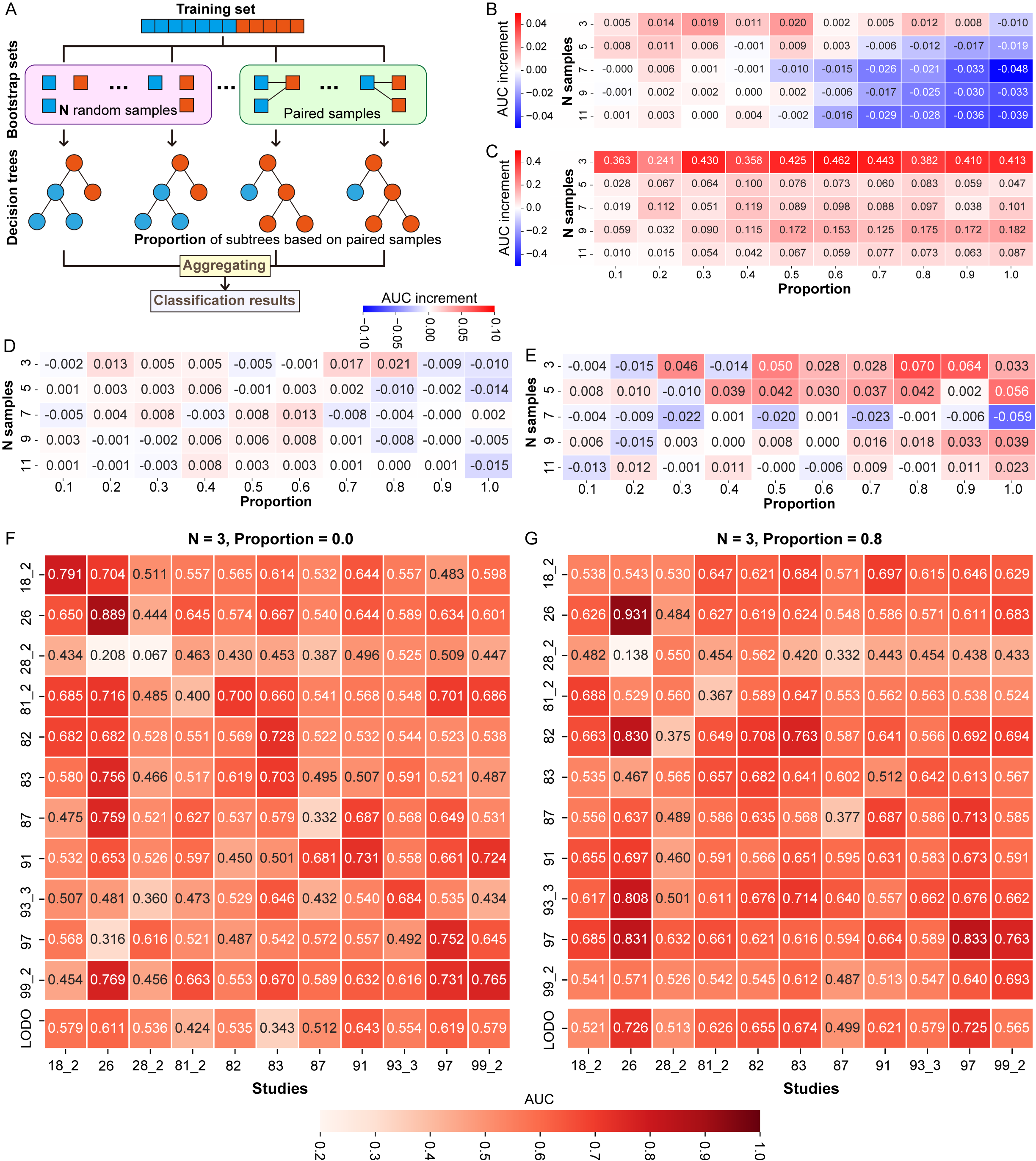


**Figure S6. Matched Sampling Random Forest (MSRF) model and its application in colorectal cancer (CRC).** (A) Flowchart of the MSRF model: random samples in a certain percentage of subtrees are replaced with matched samples. (B) The average AUC increment of disease diagnostic models of all single cohorts. Sixty-one cohorts, which contain at least 20 samples per group, were tested. (C) The best-performing cohort (cohort 37_1, impaired glucose tolerance) showed an AUC increment of up to 0.46. (D) and (E) Based on experiments of 11 CRC cohorts, the average AUC increment for MSRF models with varying proportions of subtrees (denoted as *ratio*) that used matching information is shown for (D) the study-to-study validation and (E) the leave-one-dataset-out validation, respectively. (F) AUC validation matrices for the CRC diagnostic model without any matching information. (G) AUC validation matrices for the CRC diagnostic model with 80% of the sub-trees constructed using matched samples. Detailed project information related to these models is provided in Table S3. Results for other studies can be accessed at https://github.com/ddhmed/miMatch.


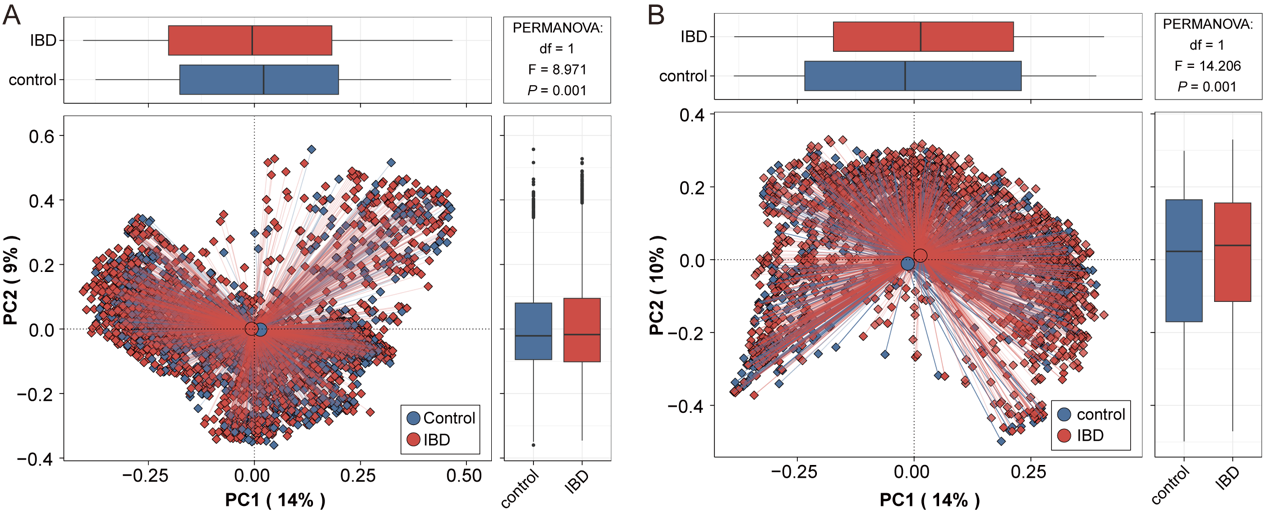


**Figure S7. Beta diversity of IBD before and after matching**. The principal coordinates analysis (PCoA) of beta diversity indicated that microbial diversity differences between cases and controls remained significant (A) before and (B) after matching. The centroids for the groups are depicted by outlined circles.
